# Sorbitol-fermenting *Escherichia coli* O157, Scotland

**DOI:** 10.3201/eid1605.091919

**Published:** 2010-05

**Authors:** Kevin G.J. Pollock, Mary E. Locking, T. James Beattie, Heather Maxwell, Ian Ramage, David Hughes, Jennifer Cowieson, Lesley Allison, Mary Hanson, John M. Cowden

**Affiliations:** Health Protection Scotland, Glasgow, UK (K.G.J. Pollock, M.E. Locking. J.M. Cowden); Yorkhill Hospital, Glasgow (T.J. Beattie, H. Maxwell, I. Ramage, D. Hughes, J. Cowieson); Scottish *E. coli* O157/VTEC Reference Laboratory, Edinburgh, UK (L. Allison, M. Hanson)

**Keywords:** Outbreak, Escherichia coli O157, sorbitol-fermenting, clinical sequelae, bacteria, Scotland, letter

**To the Editor**: Verotoxin-producing *Escherichia coli* (VTEC) of serogroup O157 causes severe gastrointestinal and renal illness; clinical signs may be mild diarrhea, hemorrhagic colitis, or hemolytic uremic syndrome (HUS). Typically, 10%–15% of reported VTEC infections quickly progress to HUS (*1*). Sorbitol-fermenting (SF)–O157 strains have emerged in continental Europe (*2*,*3*). Some evidence suggests that SF-O157 is more frequently associated with HUS than are non-sorbitol–fermenting strains (*3*–*6*). SF-O157 shows increased adherence to colonic epithelial cells and may in turn cause a more potent inflammatory host response, resulting in a higher risk for HUS (*4*). The potentially greater virulence of SF-O157 requires urgent identification of its reservoir(s) and vehicle(s) of infection, as well as determination of genetic or other predisposing factors for infection with this strain. To understand whether the host pathophysiologic responses to SF-O157 and non–SF-O157 strains differ, we analyzed a cohort of children with HUS who were infected with *E. coli* O157.

 During April and May 2006, Health Protection Scotland (HPS) identified 18 cases of verotoxin-producing SF-O157 infection in Scotland, 13 of which were associated with a nursery. HUS developed in 8 of the 18 patients; those with thrombotic microangiopathy were admitted to the renal unit of a specialist pediatric hospital, which immediately reports cases of HUS to HPS as part of national surveillance (*7*). To test the hypothesis that SF-O157 was more virulent than non–SF-O157, we performed an age-matched, nested case–case study of HUS case-patients and analyzed host clinical markers, treatment, and outcomes from SF-O157 and non–SF-O157 cases in 2006. Clinical questionnaires, patient information sheets, and consent forms were completed by clinicians for each case-patient and returned to HPS; data were entered into a database in Epi Info version 6 (Centers for Disease Control and Prevention, Atlanta, GA, USA).

 Statistical analysis by *t* test showed that nadirs for serum albumin were significantly higher for children with SF-O157 HUS (p = 0.03; Table) than for children with non–SF-O157 HUS and that children with SF-O157 HUS had significantly more sessions of hemodialysis than did children with non–SF-O157 HUS (p = 0.01; Table). All case-patients were oligoanuric; the 2 groups did not differ with respect to this parameter. Initial signs and symptoms were similar for both sets of patients, i.e., classic VTEC symptoms of bloody diarrhea and abdominal pain. This finding is in acccordance with those of other studies of SF-O157 outbreaks, which also noted signs and symptoms compatible with VTEC-associated gastroenteritis (*5*,*6*).

**Table:** Characteristics of patients infected with non–SF-O157 versus SF-O157 *Escherichia coli*, Scotland, 2006*

Characteristic	SF-O157, n = 8	Non–SF-O157, n = 19	p value
Age, y ± SEM	5.4 ± 1.4	5.1 ± 0.9	
Sign or symptom, no. (%) patients			
Diarrhea	8	19	
Bloody diarrhea	6 (75)	14 (74)	0.79
Abdominal pain	6 (75)	13 (68)	0.13
Fever	1 (12)	4 (21)	0.73
Neurologic involvement	2 (25)	4 (21)	0.82
Clinical parameter, mean ± SEM			
Anuria, d	11.7 ± 2.7	7.9 ± 1.4	0.20
Leukocyte count, ×10^9^/L	26.4 ± 2.1	36.4 ± 10.1	0.34
C-reactive protein, mg/L	65.6 ± 27.1	93.4 ± 23.1	0.31
Serum albumin, g/L	32.4 ± 7.0	23.2 ± 1.0	0.03
Lactate dehydrogenase, IU/L	2,774 ± 280	2,556 ± 324	0.68
Hospital stay, d	17.9 ± 3.7	16.1 ± 2.9	0.71
Treatment, mean no. sessions ± SEM			
Peritoneal dialysis	13.4 ± 2.3	7.4 ± 1.9	0.07
Hemodialysis	20.5 ± 3.5	9.3 ± 1.3	0.01
Outcomes, 1 y follow-up, no.	n = 6	n = 19	
Full recovery, no. patients	6	17	
Clinical sequelae, no. patients	0	2†	

*SF, sorbitol-fermenting. †1 with hypertension, 1 with abdominal pain/vomiting.

 Our study highlights a number of lessons. Medical practitioners rarely have the opportunity to recognize patients at such an appreciable and predictable risk of progressing rapidly to anuric renal failure as they do when they see children with early O157 infection. Failure to appreciate the potential gravity of O157 infection and the possible development of HUS may result in avoidable illness and even death. Our investigation of the prehospital management of SF-O157 and non–SF-O157 in this cohort found no difference in pharmacologic intervention or duration of delay in admission to hospital.

Our study has limitations. A number of patients in the cohort were prescribed antimicrobial drugs and/or antimotility drugs or were sent home from the local hospital without hospital admission or further monitoring; such actions potentially exacerbate clinical outcomes (*1*,*8*). We recognize that comparison of the SF-O157 outbreak strain with non–SF-O157 strains (some of which caused sporadic cases) may be a potential confounding factor in the analysis. However, recently published work has indicated no statistically significant differences in the verotoxin proteins encoded by SF-O157 or non–SF-O157 strains or in their level of toxicity (*9*). Other virulence factors may contribute to increased likelihood of HUS (*4*).

Our data suggest that infection with SF-O157 results in less severe colitis than does the more common non–SF-O157 infection. Less severe colitis could result in a lower risk for renal disease because less verotoxin would be translocated into the bloodstream and bound to the kidneys. However, patients infected with SF-O157 had anuria for longer periods and consequently had longer sessions of peritoneal and hemodialysis. Although unknown bacterial or host inflammatory cytokines may contribute to enhanced disease progression, this observation is surprising and requires further investigation. Additional research is needed to learn more about the virulence of SF-O157 strains and establish other host factors that contribute to disease progression.
